# Increased Left Ventricular Myocardial Extracellular Volume Assessed by Cardiac Computed Tomography as a Consequence of Aortic Stenosis and Coexisting Cardiovascular Risk Factors

**DOI:** 10.3390/jcm14134435

**Published:** 2025-06-22

**Authors:** Adrian Martuszewski, Patrycja Paluszkiewicz, Rafał Poręba, Paweł Gać

**Affiliations:** 1 Department of Environmental Health, Occupational Medicine and Epidemiology, Wroclaw Medical University, Mikulicza-Radeckiego 7, 50-345 Wrocław, Poland; pawel.gac@umw.edu.pl; 2 Department of Neurology, Specialist Hospital in Walbrzych, 58-309 Wałbrzych, Poland; 3 Department of Emergency Medical Service, Wroclaw Medical University, Bartla 5, 50-367 Wrocław, Poland; 4 Department of Biological Principles of Physical Activity, Wroclaw University of Health and Sport Sciences, 51-612 Wrocław, Poland; 5 Centre of Diagnostic Imaging, 4th Military Hospital, Weigla 5, 50-981 Wrocław, Poland

**Keywords:** extracellular matrix, myocardial fibrosis, computed tomography angiography, TAVR, ventricular remodeling, valvular heart disease, subclinical cardiac damage, cardiac risk assessment

## Abstract

**Background/Objectives**: Extracellular volume (ECV) expansion reflects myocardial fibrosis and may play a role in subjects with severe aortic stenosis (AS) receiving transcatheter aortic valve implantation (TAVI). This study aimed to assess the relationship between cardiovascular risk factors (CVRF), AS severity and left ventricular myocardial ECV measured by cardiac computed tomography (CCT). **Methods**: 61 patients qualified for TAVI underwent pre-procedural CCT. CVRFs were recorded, including advanced age, male gender, obesity, hypertension, hypercholesterolemia, hypertriglyceridemia, type 2 diabetes, and smoking. The CCT protocol included non-contrast (for aortic valve calcium score, AVCS), angiographic (for vascular access planning), and delayed phases (for left atrial appendage thrombus assessment). ECV was calculated from attenuation values of the interventricular septum and left ventricular cavity assessed in native and delayed phases. Patients were stratified based on the presence/absence of individual CVRFs, median AVCS, and aortic valve area (AVA). **Results**: Mean ECV was higher in patients with hypertension (28.01% vs. 26.93%, *p* = 0.03), smokers (28.71% vs. 26.52%, *p* = 0.01), AVCS ≥ 2975 (28.08% vs. 26.95%, *p* = 0.02), and AVA < 0.95 cm^2^ (28.63% vs. 26.53%, *p* = 0.01). Positive correlations were found between ECV and the number of CVRFs (r = 0.49, *p* = 0.01), BMI (r = 0.30, *p* = 0.01), systolic BP (r = 0.31, *p* = 0.02), and AVCS (r = 0.36, *p* = 0.01); AVA correlated negatively (r = −0.59, *p* = 0.01). Regression showed that hypertension, smoking, and smaller AVA were independent predictors of higher ECV. **Conclusions**: Among TAVI candidates, hypertension, smoking, and more advanced AS are independently associated with increased myocardial ECV on CCT. These findings may reflect subclinical myocardial remodeling and support the added diagnostic value of ECV in pre-TAVI assessment.

## 1. Introduction

Aortic stenosis (AS) is an acquired valvular disease in developed countries, with the frequency of its severe form reaching 3.4% among individuals aged ≥ 75 years [1]. The aging of the population leads to a systematic increase in the global burden of this condition, resulting in a growing demand for valve interventions [2]. It is noteworthy that transthyretin cardiac amyloidosis (ATTR-CA) frequently coexists with aortic stenosis in older patients, especially those undergoing transcatheter aortic valve implantation (TAVI), with reported prevalence ranging from 4% to 16% [3]. This coexistence complicates the clinical management, as amyloid deposition can accelerate valvular degeneration and significantly influence prognosis. Echocardiography remains the diagnostic standard, though in ambiguous cases, a multimodal approach involving cardiac magnetic resonance (CMR) and cardiac computed tomography (CCT) is increasingly adopted [4]. Additionally, technetium-99m (99mTc)-labelled cardiac scintigraphy using agents such as 99mTc-pyrophosphate (PYP), 99mTc-3,3-diphosphono-1,2-propanodicarboxylic acid (DPD), or 99mTc-hydroxymethylene diphosphonate (HMDP) may be utilized to enhance diagnostic sensitivity for ATTR-CA, although endomyocardial biopsy remains the definitive diagnostic method, traditionally considered the gold standard [3,5,6,7,8].

Transcatheter aortic valve implantation has become the reference procedure for individuals at high or intermediate surgical risk, offering survival outcomes comparable to surgical aortic valve replacement (SAVR) [9]. Recent data from the PARTNER 3 randomized trial demonstrated the superiority of TAVI over SAVR, even in patients at low surgical risk. The TAVI group showed lower rates of the composite endpoint of stroke, death, and rehospitalization, as well as reduced risk of stroke and atrial arrhythmias [10].

Multidetector CCT plays a key role in patient selection, allowing precise assessment of annular dimensions, extent of calcification, and vascular access anatomy [11,12,13]. According to the ESC/EACTS guidelines, precise imaging-based quantification of aortic valve calcium score (AVCS) and left ventricular remodeling parameters, including myocardial fibrosis, is essential for accurate risk stratification and optimal timing of intervention in patients with severe AS [14]. CCT reconstructions also help determine optimal fluoroscopic projections, reducing procedure time and periprocedural complications [15,16,17].

Classical cardiovascular risk factors (CVRF) – arterial hypertension (AH), dyslipidemia, diabetes mellitus (DM), obesity, and smoking – remain key contributors to structural heart damage and cardiovascular events [18]. The cumulative toxic effects of smoking and metabolic disorders promote earlier onset of hypertension and accelerate coronary artery disease (CAD) progression [19].

Population studies show that the greater the number of coexisting risk factors, the higher the mortality and incidence of cardiovascular events [20]. Particularly, the combination of smoking and obesity in hypertensive patients significantly raises the risk of cardiac complications beyond the individual effects of each factor [21,22].

Expansion of the extracellular volume (ECV) reflects diffuse myocardial fibrosis, a substrate for heart failure (HF), arrhythmias, and increased mortality [23,24,25]. Although T1 mapping on CMR remains the gold standard for ECV measurement, late-phase CT provides a validated, histologically confirmed alternative [26,27,28].

In patients with severe AS, high ECV values correlate with more advanced extra-valvular cardiac damage and worse outcomes after TAVI [29]. Despite the growing number of studies on ECV imaging using CCT and CMR, the impact of classical CVRFs—such as AH, DM, or dyslipidemia—on ECV remains incompletely understood and warrants further investigation [30,31,32,33,34].

The aim of our study was to assess the relationship between the presence of established CVRFs, the severity of AS, and ECV of the left ventricular myocardium as measured in routine pre-TAVI CCT protocols.

## 2. Materials and Methods

### 2.1. Study Design and Ethical Considerations

We conducted a retrospective observational study at a single center, titled: “Serum galectin-3 concentration and myocardial extracellular volume assessed by computed tomography as potential markers of cardiovascular health”. The study protocol adhered strictly to the principles of the Declaration of Helsinki and received approval from the Institutional Review Board of Wroclaw Medical University (approval no. 1023/2021, dated 13 December 2021). Each participant provided written informed consent prior to inclusion.

### 2.2. Study Population and Clinical Data Collection

Group size was determined using a sample size calculator. The selection conditions were as follows: population size 3 million, fraction size 0.1, maximum error 10%, confidence level 95%. The required minimum size of the study group was 35. Sixty-one patients were studied. Hence, according to the sample size calculator, the maximum error is 8%.

Participants were eligible for inclusion if they met the following criteria: age > 18 years, diagnosis of aortic stenosis, qualified for treatment with TAVI, and consent to participate in the study. The exclusion criteria were: previous myocardial infarction; prior PCI or cardiac surgery (including valve surgery and CABG) or other vascular surgery; previously diagnosed non-ischemic myocardial injury with late gadolinium enhancement (e.g., in hypertrophic cardiomyopathy, amyloidosis, sarcoidosis, dilated cardiomyopathy, or myocarditis); contraindications to iodinated contrast agents; and insufficient technical quality of the computed tomography images.

The study involved 61 consecutive adult patients scheduled for TAVI who were capable of providing informed consent. We collected demographic data, anthropometric measurements, and clinical information retrospectively from electronic medical records. Body mass index (BMI) classification followed World Health Organization guidelines (BMI ≥ 30 kg/m^2^ for obesity, ≥25 kg/m^2^ for overweight) [35]. AH was diagnosed according to European Society of Hypertension criteria [36], hypercholesterolemia and hypertriglyceridemia followed ESC recommendations (LDL-C 115 mg/dL [≥3.0 mmol/L]; triglycerides 150 mg/dL [≥1.7 mmol/L]) [37], and type 2 DM was identified based on current national diabetes standards [38]. Smoking status and additional medical history details were obtained directly during clinical interviews with patients. For statistical purposes, cardiovascular risk factors were also analyzed as a cumulative score. The CVRF score was calculated as the total number of the following risk factors present in each patient: age > 75 years, male sex, obesity (BMI ≥ 30 kg/m^2^), arterial hypertension, hypercholesterolemia (LDL ≥ 115 mg/dL), hypertriglyceridemia (≥150 mg/dL), type 2 diabetes mellitus, and active or former smoking. Each factor was scored as one point if present, resulting in a total score ranging from 0 to 8.

**Figure 1:**
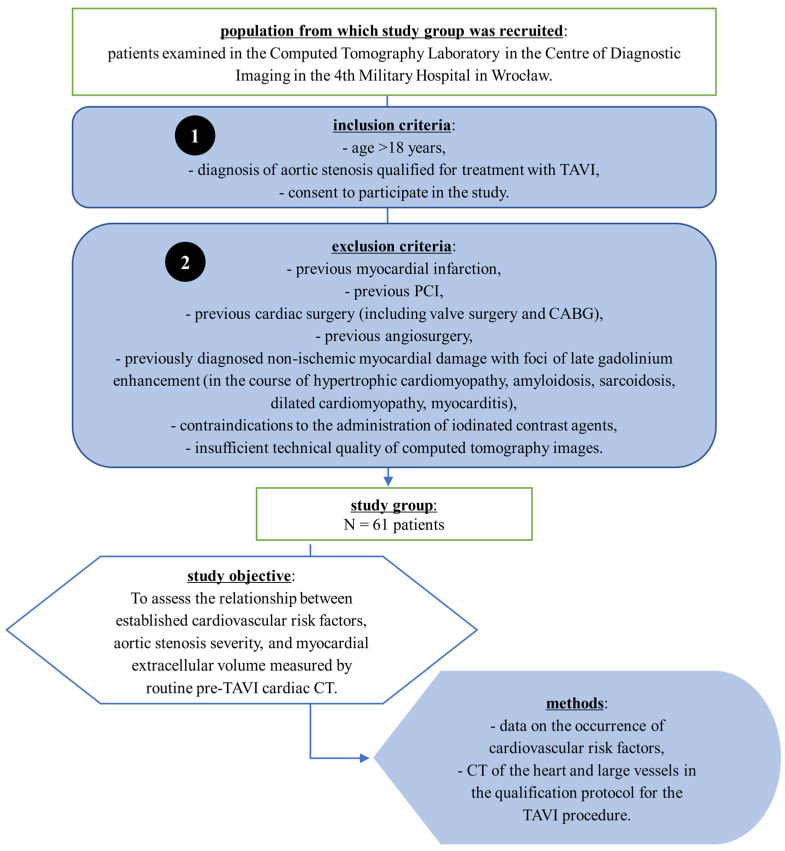
Flowchart of patient selection and study design. Patients with severe aortic stenosis who were qualified for transcatheter aortic valve implantation (TAVI) were enrolled in the CT Laboratory of the 4th Military Hospital in Wrocław. Inclusion and exclusion criteria are listed. The study group consisted of 61 patients. The objective was to assess the relationship between cardiovascular risk factors, aortic stenosis severity, and myocardial extracellular volume (ECV) measured by pre-procedural cardiac CT. Data were obtained from routine cardiovascular risk assessment and computed tomography scans performed as part of the TAVI qualification protocol. Abbreviations: CABG, coronary artery bypass grafting; CT, computed tomography; PCI, percutaneous coronary intervention; TAVI, transcatheter aortic valve implantation.

A flow chart illustrating the patient selection process is presented in Figure 1.

### 2.3. Cardiac Computed Tomography Protocol

All participants underwent CCT using a dual source 384-slice CT scanner SOMATOM Force (Siemens Healthcare, Erlangen, Germany) and our institution’s standard pre-TAVI protocol. The protocol consisted of several phases: an initial topogram, a non-contrast (native) phase to quantify AVCS, an arterial phase to assess valve anatomy and vascular access, and a delayed phase performed three minutes after contrast injection, specifically to rule out thrombus in the left atrial appendage.

Tube voltage was adapted to the requirements of each acquisition phase. Both the non-contrast (native) and delayed phases were acquired at 120 kV to optimize tissue characterization and allow for ECV quantification. The angiographic (arterial) phase was performed at 100 kV to enhance vascular contrast and reduce radiation dose, which is in line with current clinical practice. Images were reconstructed as 3.0 mm thick axial slices for the native and delayed phases and as 0.6 mm thick axial slices for the arterial phase.

Dual-energy acquisitions were not employed, as our standard protocol favors single-energy acquisitions at 120 kV. This approach optimizes workflow, maintains consistency across examinations, and provides sufficient diagnostic information without added complexity or increased radiation exposure.

The delayed acquisition at three minutes post-contrast injection was chosen to allow for adequate washout from the left ventricular cavity, which improves differentiation between the blood pool and potential thrombus. This timing is supported by prior clinical protocols [39,40,41].

Intravenous contrast was administered as Iohexol (Omnipaque 350, GE Healthcare A.S., Nycoveien 1, NO-0485 Oslo, Norway; concentration: 350 mg I/mL) at a dose of 70–100 mL, with an injection rate of 4 mL/s. At our institution, cardiac CT imaging prior to TAVI is routinely performed with Iohexol (Omnipaque 350) because of its reliable enhancement and favorable safety profile. The total contrast volume (70–100 mL) was chosen to ensure sufficient enhancement of the myocardium and LAA for reliable thrombus exclusion and to provide optimal visualization of all potential vascular access routes (including upper and lower limb arteries). This variability in contrast volume accounts for patient-specific factors such as body weight and cardiac output and is consistent with current clinical standards [17]. It is also critical for obtaining high-quality delayed-phase myocardial enhancement necessary for ECV analysis.

The injection rate of 4 mL/s is routinely used at our institution, providing an optimal balance between adequate vascular enhancement, consistent myocardial opacification, and patient safety, minimizing risks such as extravasation or contrast-induced nephropathy. Previous studies have confirmed good diagnostic quality at similar injection rates [28].

### 2.4. Image Interpretation and Analysis

All images were evaluated by a cardiovascular radiologist with many years of experience, holding multiple credentials: the European Diploma in Radiology, the European Board of Cardiovascular Radiology Diploma, the Polish Society of Radiology Cardiovascular Certification, and the European Association of Cardiovascular Imaging (EACVI) Exams in Cardiac CT and CMR.

AVCS was assessed on native-phase images using the syngo.CT CaScoring software (part of the syngo.via platform, version VB60B_HF02; Siemens Healthineers AG, Forchheim, Germany). The analysis included only calcifications localized to the valve cusps or annulus, identified using the standard Agatston threshold of >130 HU. A second radiologist independently verified each segmentation, and any discrepancies were resolved through consensus.

We measured the aortic valve area (AVA) using direct planimetry. Multiplanar reconstructions at mid-systole, perpendicular to the valve plane, were generated to achieve an accurate cross-sectional view of the valve. The smallest valve opening was manually delineated at the leaflet tips.

**Figure 2:**
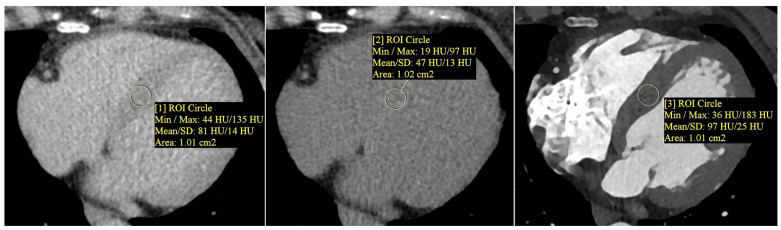
Measurement of myocardial and blood pool attenuation (Hounsfield units, HU) was performed by placing circular regions of interest (ROIs, about 1.0 cm^2^) on native and delayed-phase cardiac CT images to quantify extracellular volume (ECV). From left to right: native (non-contrast) myocardium—ROI [1]; delayed-phase myocardium—ROI [2]; delayed-phase blood pool—ROI [3]. ROIs were positioned to avoid trabeculae, epicardial fat, and calcifications. Mean HU values ± SD are displayed. ECV was calculated using a standard formula that incorporates the patient’s hematocrit and the attenuation difference between the myocardium and blood pool. Note: ROI numbers [1], [2], and [3] refer to the image annotations and do not indicate literature references.

**Figure 3:**
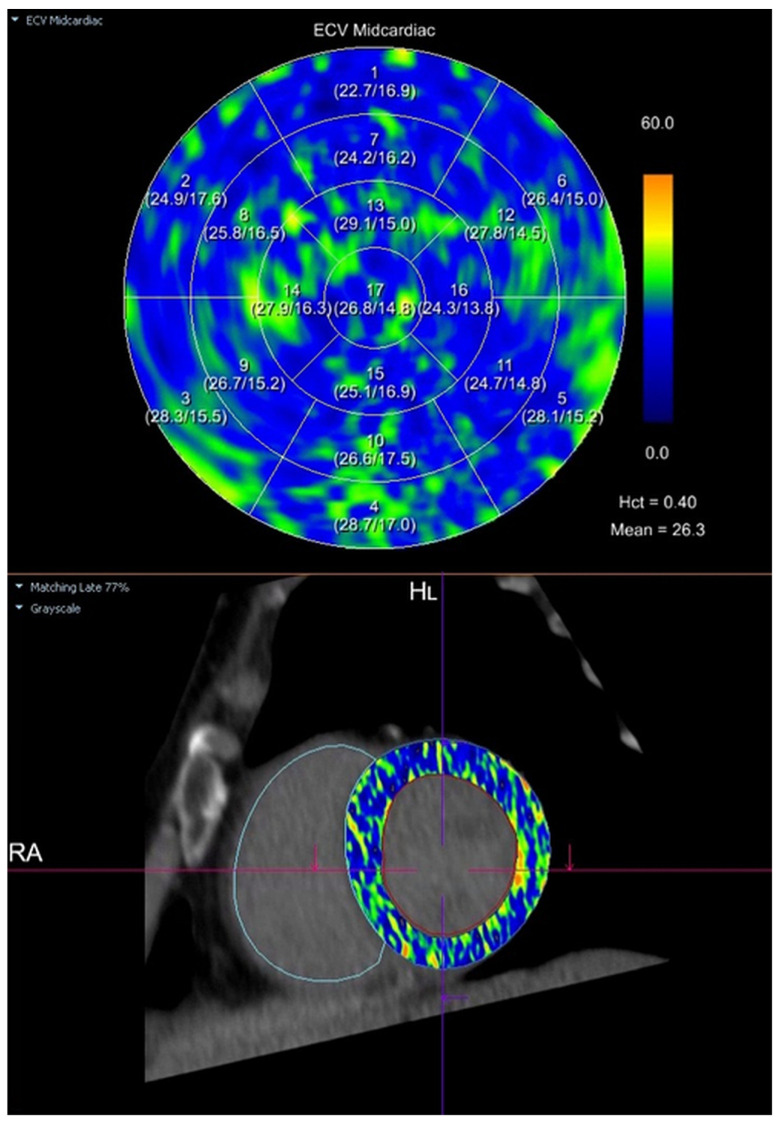
Semi-automatic quantification of myocardial extracellular volume (ECV) using cardiac computed tomography (CT). The ECV map was generated in the mid-ventricular short-axis plane using the CT Cardiac Functional Analysis post-processing application (Siemens Healthineers). The upper panel presents a polar (bullseye) plot with segmental ECV values across the standardized 17-segment model of the left ventricular myocardium. The lower panel shows the corresponding mid-ventricular short-axis image with the ECV parametric map superimposed. ECV was calculated based on pre-contrast and delayed-phase images, with a hematocrit (Hct) of 0.40. The mean global myocardial ECV was 26.3%. Legend: A polar (bullseye) plot (upper panel) displays regional myocardial ECV values for each of the standardized 17 segments of the left ventricle. Segmental ECV values are color-coded according to the adjacent scale, where blue indicates lower ECV values and yellow-orange indicates higher ECV values (range: 0–60%). The lower panel shows the corresponding mid-ventricular short-axis CT image, with the ECV parametric map superimposed onto the myocardium, illustrating the spatial distribution of myocardial ECV.

Myocardial extracellular volume (ECV) was calculated semi-automatically using the CT Cardiac Functional Analysis post-processing application (module version 3.0.1, running on syngo.via platform version VB60B_HF02; Siemens Healthineers AG, Forchheim, Germany)—Figure 2. The calculation was based on native and delayed phase images, and an ECV map of the left ventricle was generated, divided into 17 standard myocardial segments (LV). The final ECV value used for analysis represented the mean of all 17 segments (global myocardial ECV)—Figure 3.

The software applies the following validated formula:

$$myocardial\;ECV\;[\%]=\left(1-hematocrit\right)\times \frac{{∆HU}_{myocardium}}{{∆HU}_{blood}}$$

where ΔHU represents the change in attenuation (in Hounsfield Units) between native and delayed acquisitions. The hematocrit was obtained from blood samples collected on the day of CT.

Inter- and intra-observer variability of ECV measurements was assessed in a random sample of 20 patients, showing high reproducibility (intra-class correlation coefficient, ICC > 0.90). Detailed methodological validation can be found in prior studies [42,43,44,45].

### 2.5. Statistical Methods

Data were analyzed using the Statistica software version 14.0.0.15. Normal distribution was verified for all continuous variables using the Shapiro–Wilk test. Continuous data, including ECV, are described as mean ± standard deviation. Pearson correlation analysis was used to evaluate linear relationships between quantitative variables. Group comparisons were made for independent samples using the Student’s *t*-test. To determine independent predictors of myocardial ECV, we performed a backward stepwise multiple linear regression analysis (ordinary least squares method). Statistical significance was set at a two-tailed *p*-value below 0.05.

### 2.6. Post-Hoc Statistical Power Analysis

We conducted a post-hoc power analysis in R (version 4.4.2, using the *pwr* package [46]), with α set at 0.05. The power analysis indicated very high statistical power for significant correlations observed in our study: between ECV and aortic valve area (r = −0.59; power = 0.999), number of cardiovascular risk factors (r = 0.49; power = 0.984), and AVCS (r = 0.36; power = 0.824). However, weaker correlations (e.g., systolic BP: r = 0.31, power = 0.691; BMI: r = 0.30, power = 0.661) did not reach conventional power thresholds, indicating a risk for type II error. We estimated that a sample size of approximately 130–138 individuals would be necessary to achieve a power of 0.95 for correlations of such magnitude. The multiple regression model, incorporating AH, smoking, and AVA, explained 51.9% of the variance in ECV (adjusted R^2^ = 0.519), with an estimated power of 0.99, confirming its strong predictive value. In line with Cohen’s guidelines [47], we performed this power analysis post-hoc to ensure our significant results were based on statistically robust foundations.

## 3. Results

The study enrolled 61 consecutive candidates for TAVI. The cohort was elderly (79 ± 9 years) and predominantly male (62%). AH and hypercholesterolemia were the most frequent cardiovascular risk factors, each affecting just over half of the group. AH occurred in 54% of patients, hypercholesterolemia in 57%, current or former smoking in 46%, hypertriglyceridemia in 33%, and DM in roughly 10% of the cohort. Baseline characteristics are summarized in Table 1.

Contrast-enhanced CCT confirmed severe, calcific tricuspid AS in almost every participant. Only two bicuspid valves were identified. The mean AVCS exceeded 3300 Agatston units, and the average AVA was just under 1 cm^2^. On average, the annulus measured about 24 × 27 mm, the Sinuses of Valsalva were roughly 32 mm wide, and their height was close to 21 mm. Left-ventricular ECV measured on delayed images averaged 27.5 ± 1.9%. Table 2 presents aortic valve assessment parameters and left ventricular myocardial ECV evaluated by multidetector CCT prior to TAVI in the study population.

**Figure 4:**
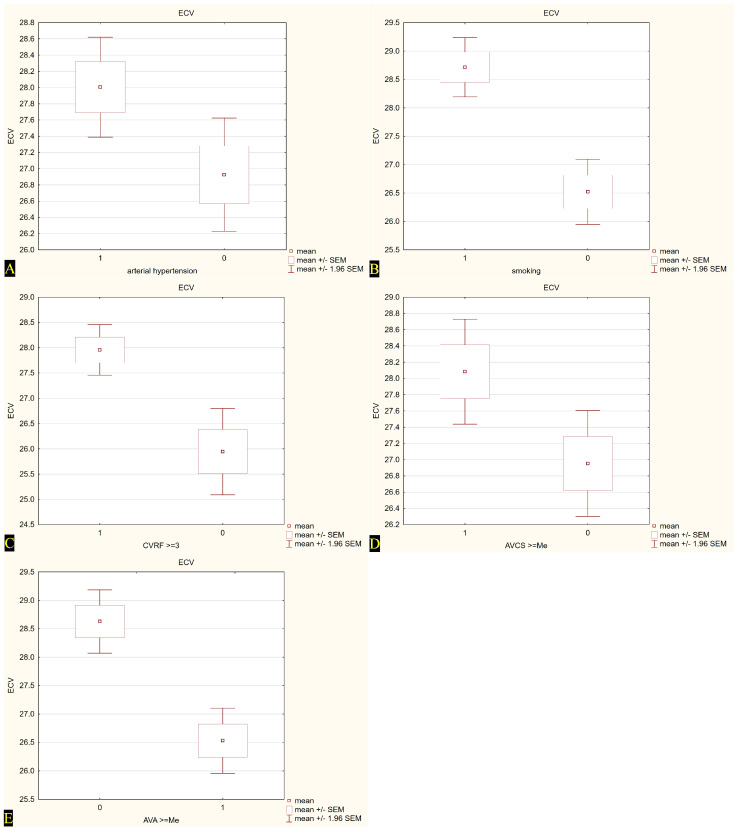
Extracellular volume (ECV) values in relation to selected cardiovascular risk factors and calcium scores. (**A**) ECV in patients with (1) and without (0) arterial hypertension; (**B**) ECV in smokers (1) and non-smokers (0); (**C**) ECV in subjects with ≥3 cardiovascular risk factors (CVRF) (1) vs. <3 CVRF (0); (**D**) ECV in patients with aortic valve calcium score (AVCS) ≥ median (1) vs. <median (0); (**E**) ECV in patients with aortic valve area (AVA) < median (0) vs. ≥median (1). Mean values are presented with standard error of the mean (±SEM) and 95% confidence intervals (±1.96 SEM).

When the population was dichotomized at conventional or median cut-points, we saw higher ECV values in patients with AH (*p* = 0.03) and in current or former smokers (*p* = 0.01). Notably, ECV also increased across the upper halves of AVCS and CVRF burden, whereas it was lower among individuals with a larger AVA (*p* < 0.05). No significant differences emerged with respect to age, gender, obesity, dyslipidemia, diabetes, or hypertriglyceridemia. Across the whole sample, a stepwise rise in ECV accompanied the accumulation of risk factors: subjects carrying at least three risk factors showed a mean ECV roughly two percentage points higher than those with fewer than three (*p* = 0.01). Valve-related disease severity mirrored myocardial involvement. Patients with an AVA below the median (<0.95 cm^2^) exhibited markedly greater ECV than those above it, while a higher AVCS was likewise associated with expanded extracellular space. Table 3 and Figure 4 summarize myocardial ECV as assessed by CCT before TAVI across the analyzed patient subgroups.

Correlation analysis demonstrated positive, moderate relations of ECV with BMI, systolic BP, overall risk-factor count, and AVCS (r = 0.30–0.49, *p* ≤ 0.02). Importantly, ECV was strongly and inversely correlated with AVA (r = −0.59, *p* < 0.01). Other laboratory and hemodynamic variables were not significantly correlated. Table 4 presents the results of Pearson’s correlation analysis between pre-procedurally assessed myocardial ECV and other quantitative parameters in the study cohort.

Our multivariable model confirmed that AH and smoking independently predicted higher ECV, while a larger AVA predicted lower ECV. Together, these factors accounted for just over half of the variance in myocardial ECV (adjusted R^2^ about 0.52; overall model *p* < 0.001). Table 5 summarizes the results of multiple linear regression identifying independent determinants of increased myocardial ECV in the study population.

All cardiovascular risk factors, as well as quantitative parameters of the aortic valve assessed by CT, were included in the multivariable regression model as potential contributors to ECV. This approach enabled the identification of independent predictors while accounting for the mutual relationships between all analyzed variables.

In summary, we found that myocardial ECV was highest in patients with AH or a history of smoking and in those with more advanced AS. Notably, ECV rose stepwise with each additional cardiovascular risk factor.

## 4. Discussion

In our cohort of 61 elderly TAVI candidates in their late seventies to early eighties, left-ventricular ECV averaged 27.5 ± 1.9%, well above reference values for healthy myocardium. Hypertension and a history of smoking each nudged that figure up by roughly one to two percentage points (28.0% vs. 26.9% for AH, 28.7% vs. 26.5% for smokers), and both remained independent predictors of a larger ECV alongside a smaller AVA, the three together explaining just over half of the variance in fibrotic burden (adjusted R^2^ ≈ 0.52). We also saw a clear dose–response effect: ECV climbed with every additional conventional risk factor and tracked the AVCS while it fell as the valve orifice widened. Taken together, these findings suggest that CT-derived ECV captures the cumulative hit of classic CVRFs and the hemodynamic stress of advanced stenosis in real-world TAVI candidates. Since this study exclusively involved patients with severe aortic stenosis, caution is required in extrapolating these findings to broader populations. Additional research across more diverse clinical contexts is needed to evaluate the generalizability of our results.

### 4.1. AVA-ECV Relationship

Our analysis demonstrates a clear inverse relationship between AVA and myocardial ECV in patients with severe AS. Patients with smaller valve areas consistently logged higher ECV values, a straightforward signal that the ventricle lays down extra collagen as the orifice narrows. This finding aligns with prior observations that patients with severe AS who develop pronounced myocardial fibrosis tend to have markedly reduced valve areas [48]. The connection is pathophysiologically plausible: a smaller AVA imposes greater pressure overload on the left ventricle over time, promoting interstitial collagen deposition and raising ECV. It is noteworthy, however, that AS severity alone may not fully dictate fibrotic burden—individual variation and disease duration also modulate myocardial response. We, therefore, interpret the elevated ECV in low-AVA patients as reflecting the cumulative impact of chronic afterload stress, consistent with but not solely determined by the anatomic severity of stenosis. This interpretation avoids overreach, recognizing that while our results concur with the literature on severe AS, fibrosis development can vary among patients with similar valve areas.

### 4.2. AVCS-ECV Relationship

Our study also found that a higher AVCS was associated with increased myocardial ECV. Individuals with heavily calcified valves (AVCS above the median) had significantly greater ECV expansion than those with less calcification. This result is in line with the understanding that extensive valvular calcification signifies advanced degenerative AS, which likely corresponds to a longer-standing disease process and more time for diffuse myocardial fibrosis to accumulate [49]. Fibrosis builds in the lockstep with valve calcification. Published data support this parallel: for example, one imaging study [48] noted that severe AS patients with evidence of myocardial fibrosis tended to have higher calcified valve volumes. The mentioned study found that calcification was not an independent predictor of fibrosis once other severity factors were considered, suggesting that calcific burden and fibrotic burden both reflect the underlying severity of the disease rather than a direct causal link. Our findings agree with this nuance; —valve calcification itself correlates with myocardial ECV because both are downstream consequences of prolonged valve pathology. Moreover, recent research outcomes underscore the interplay of these factors, showing that the extent of AVCS and the degree of myocardial fibrosis each independently contribute to prognosis after TAVI [50]. Thus, our observation of elevated ECV in high-AVCS patients dovetails with literature indicating that calcific severity and myocardial damage often go hand in hand yet represent distinct aspects of AS progression. We refrain from overinterpreting causality, but our data strongly support that a heavily calcified valve is a marker for a heart that has sustained greater fibrotic injury.

### 4.3. Aortic Stenosis Severity Assessed by Other Parameters and Its Relationship to ECV

Beyond AVA and AVCS, we considered AS severity using other clinical parameters and compared our results to findings in the literature. We did not directly measure transvalvular gradients in our analysis, but prior studies suggest that the relationship between conventional hemodynamic severity indices and diffuse fibrosis is not straightforward. For instance, one CMR study [51] reported that the myocardial ECV did not vary significantly with AS severity defined by peak aortic jet velocity (*p* = 0.30). This implies that two patients with similar high gradients can exhibit quite different degrees of interstitial fibrosis. On the other hand, certain AS subtypes with impaired forward flow show a disproportionate fibrotic response. In particular, patients with classical low-flow, low-gradient AS (often characterized by left ventricular dysfunction and paradoxically lower gradients despite severe stenosis) have been shown to harbor higher diffuse fibrosis burdens than those with high-gradient AS. In one series, the low-flow, low-gradient group exhibited significantly elevated ECV and greater scar volume compared to normal-flow, high-gradient patients [52]. These contrasting observations highlight that myocardial fibrosis in AS is influenced by more than just instantaneous measures of stenosis severity. Factors such as flow status, ventricular compensatory capacity, and perhaps chronicity of pressure overload all contribute. Our findings that ECV was elevated in patients with smaller AVA (and higher AVCS) reinforce that structural severity does play a role. However, we acknowledge in light of the literature that standard severity metrics do not perfectly predict fibrotic remodeling in every case. This perspective is cautious: we emphasize that while a tighter valve and heavy calcification generally meant more fibrosis in our cohort, exceptions can occur, especially in atypical presentations of AS.

### 4.4. Hypertension-ECV Relationship

AH emerged as a significant determinant of myocardial ECV in our study, and this observation is strongly supported by previous research. We found that patients with a history of AH had a roughly 1% higher absolute ECV on average than normotensive patients, and hypertension remained an independent predictor of elevated ECV in multivariable analysis. This finding accords with the well-established link between chronic high afterload and diffuse myocardial fibrosis. Long-standing hypertension induces left ventricular hypertrophy and stimulates fibroblast activation, leading to the accumulation of interstitial collagen. T1-mapping consistently flags more diffuse fibrosis in hypertensive hearts. For example, preliminary CMR investigations noted higher myocardial ECV in individuals with AH compared to normotensive controls (*p* > 0.05), particularly in those who have developed left ventricular hypertrophy [53]. These results mirror our experience in TAVI candidates: even in the context of severe AS, concomitant hypertension appeared to “nudge” the fibrotic burden higher. In practical terms, this suggests that AH and AS have additive deleterious effects on the myocardium. Our discussion remains measured; we interpret the hypertension effect as contributory rather than proof of causation, yet the consistency with prior studies lends confidence that the association is real. Notably, our hypertensive patients’ ECV was still within a range seen in severe AS, but the incremental rise fits the paradigm of hypertensive heart disease compounding AS-related fibrosis. This agreement with the literature strengthens the plausibility of our findings and helps validate CT-derived ECV as a marker capturing the cumulative impact of multiple pressure overload factors.

### 4.5. Smoking-ECV Relationship

Similarly, a history of smoking was linked with higher myocardial ECV in our cohort. We observed that former or current smokers had significantly greater ECV percentages than never-smokers, and smoking status was an independent predictor of diffuse fibrosis burden. This finding is in line with emerging evidence that tobacco exposure contributes to myocardial remodeling. While smoking is associated with coronary artery disease and macrovascular effects, there is growing recognition of its impact on the myocardium’s microstructure. In a recent study [54] of diabetic patients, active smokers showed markedly elevated ECV compared to non-smokers, indicating more diffuse fibrosis in the smoking group. Our findings, in an elderly, predominantly ex-smoker population awaiting TAVI, align with the notion that chronic smoking induces long-lasting fibrotic changes. The mechanism is not fully delineated, but cigarette smoke promotes systemic inflammation and oxidative stress, which can stimulate myocardial fibroblasts and collagen deposition over time. The literature on smoking and diffuse fibrosis is relatively limited. Therefore, our study adds to this area by highlighting smoking as a factor linked to higher ECV, even in patients with severe valvular disease. We interpret this cautiously: smoking history likely serves as a marker of cumulative cardiovascular injury rather than a direct cause of fibrosis, yet the congruence with prior data in other populations (e.g., smokers with diabetes) supports the credibility of this association. In summary, our results reinforce that smoking confers an added fibrotic burden on the heart, which agrees with published findings and provides further impetus for risk factor modification in AS patients.

### 4.6. BMI-ECV and Obesity-ECV Relationships

The relationship between BMI and myocardial ECV in our study was nuanced, revealing a discrepancy between BMI as a continuous variable and obesity as a categorical variable may depend on the interplay of obesity severity, duration, and metabolic health. In univariate analysis, we identified a modest positive correlation between BMI and ECV (increased BMI tending to associate with higher ECV), but when we stratified patients into obese vs. non-obese groups by the standard BMI cutoff, their mean ECV did not differ significantly. This finding requires careful interpretation. Some studies have indeed found that obesity is associated with increased diffuse myocardial fibrosis. For instance, one investigation [55] reported that overweight/obese individuals can exhibit substantially higher myocardial ECV fractions. In the study, healthy volunteers had a median ECV of about 26%, whereas obese or diabetic subjects showed a median of around 33%. Such data suggest that greater adiposity, especially when combined with metabolic disease, may promote fibrotic remodeling.

On the other hand, other research points to a more complex or even opposite effect in certain contexts. A recent CMR study [56] in young adults with early-stage obesity found lower myocardial ECV in obese patients compared to those with normal BMI. The authors postulated that in early or mild obesity, cardiomyocyte hypertrophy predominates as a remodeling mechanism without a parallel expansion of extracellular collagen. Only with chronic obesity and accompanying factors might ECV begin to rise. These seemingly conflicting findings provide important context for our results. The significant correlation we saw with continuous BMI implies a graded relationship. Each incremental gain in BMI may contribute to a small fibrosis increment, but the lack of a clear dichotomous difference suggests that a simple obesity threshold does not capture this relationship. In light of the literature, we remain cautious not to overstate the BMI-fibrosis link. Our findings generally align with reports that extreme or long-standing adiposity can exacerbate diffuse fibrosis, yet we also recognize the discrepancy as a reminder that obesity’s impact on the myocardium can vary. Thus, while we observed a trend of higher ECV with higher BMI, the evidence suggests this relationship is not absolute and may depend on the interplay of obesity severity, duration, and metabolic health.

### 4.7. Age, Gender, Dyslipidemia, and Diabetes: Non-Significant ECV Associations in Our Cohort

Finally, we did not find significant associations between myocardial ECV and certain other factors—namely age, gender, dyslipidemia, or type 2 DM—and this absence of effect is largely supported by existing studies. The lack of an age correlation in our elderly cohort is consistent with some imaging data indicating that diffuse fibrosis (as reflected by ECV) does not necessarily increase in step with age, at least beyond middle adulthood. While lifelong accumulation of collagen might intuitively raise ECV in older hearts, one study of T1 mapping in healthy subjects found no linear relationship between age and myocardial ECV [57]. It appears that by the seventh or eighth decade (the age of most TAVI patients), other factors like pressure overload or comorbidities overshadow any minor age-related fibrosis changes. Similarly, we observed no difference in ECV between male and female patients. Authors have reported subtle gender differences in diffuse fibrosis. For example, females with severe AS have been noted to have slightly higher ECV than men in certain contexts, potentially owing to hormonal or extracellular matrix differences. However, other analyses have found minimal or no gender-based variation in ECV. Our results fall in line with the latter, suggesting that gender by itself is not a major determinant of fibrotic burden once disease factors are accounted for. With respect to dyslipidemia, our study tracked both hypercholesterolemia and hypertriglyceridemia and found no significant impact on ECV. This, too, is corroborated by literature: patients with lifelong elevations in cholesterol (such as those with familial hypercholesterolemia) do not show increased myocardial ECV in the lack of overt coronary disease. In the Cholcoeur study [58], asymptomatic hypercholesterolemic patients had ECV values indistinguishable from controls, indicating that high cholesterol alone does not drive diffuse myocardial fibrosis. These findings support our interpretation that dyslipidemia’s cardiovascular effects are more vascular (plaque formation) than myocardial unless ischemic injury supervenes. Lastly, we found no significant association between DM and ECV in our TAVI cohort. This might seem surprising given the concept of diabetic cardiomyopathy, but it is actually in line with some imaging studies. For instance, a cardiovascular CMR analysis reported no significant difference in diffuse fibrosis between individuals with DM and those without [59,60]. It appears that in the absence of other factors like ischemia or advanced heart failure, well-controlled diabetes may not uniformly elevate myocardial ECV. In our population, many diabetics were likely managed and without severe cardiomyopathic changes, which could explain the neutral finding. It is worth noting that when diabetes coexists with other insults (e.g., smoking or ischemic heart disease), a synergistic fibrotic effect can emerge [54], but diabetes per se did not register as a predictor in our analysis. In summary, the lack of ECV associations for age, gender, lipid disorders, and diabetes in our study is supported by the broader evidence base. We interpret these null findings cautiously yet confidently, understanding that they reduce the likelihood of confounding in our primary results. By aligning with published data that show these variables have either minimal or context-dependent influence on myocardial fibrosis, we avoid overinterpreting any spurious links. Our discussion, thus, emphasizes the meaningful relationships (AVA, AVCS, hypertension, smoking, BMI trend) and appropriately contextualizes the non-significant factors as congruent with existing knowledge. Each of these comparisons to the literature reinforces that our findings, whether positive or negative, are credible and grounded in current scientific understanding, lending our study’s conclusions greater robustness.

### 4.8. Limitations, Strengths, and Future Directions

This retrospective, single-center analysis inevitably inherits selection bias and lacks longitudinal outcomes, yet the homogeneous TAVI cohort helps control for clinical heterogeneity. We acknowledge that the relatively small sample size (*N* = 61) limits statistical power, particularly to detect subtle effects or interactions among cardiovascular risk factors. Larger prospective studies would enhance robustness. The main strength of our study is the integration of routine CCT data with a rigorously validated ECV protocol, producing results that are directly transferable to everyday pre-TAVI workflows without additional scan time or radiation exposure. Future work should prospectively track ECV changes after valve replacement and expand enrollment across multiple centers to examine gender- and ethnicity-specific patterns and to clarify whether blood pressure and smoking cessation translate into measurable regression of diffuse fibrosis. Furthermore, future studies should assess myocardial ECV changes across different stages of cardiovascular–kidney–metabolic syndrome to better understand their combined impact on myocardial structure and function. Incorporating coronary artery calcium measurements will also be important to clarify the independent contributions of coronary atherosclerosis to myocardial extracellular volume, especially in smokers’ generalizability of these findings. Although 61 participants may appear modest, the post-hoc power analysis showed >0.80 power for the main correlations and nearly 0.99 for the multivariable model, indicating that the sample was sufficient to detect the effect sizes observed. Nonetheless, with three predictors and a modest *n*, the multivariable model may be over-fitted. It seems that external validation in a new cohort will be essential. We cannot rule out unseen confounders like circulating fibrosis markers, yet tight imaging protocols and thorough phenotyping help blunt that concern. Given that all included patients had severe AS, distinguishing the isolated effects of individual cardiovascular risk factors on myocardial remodeling was challenging due to potential overlap and interaction effects. Although patients with suspected or overt cardiac amyloidosis were excluded based on typical features of cardiac MRI, we did not perform specific screening for subclinical amyloidosis using dedicated nuclear imaging or biopsy. Therefore, the potential presence of undetected amyloid deposition remains a limitation, as it could influence myocardial ECV values independently of AS. Additionally, our study did not stratify patients according to cardiovascular–kidney–metabolic syndrome stages, which might have provided further insights into the interplay between metabolic, renal, and cardiovascular alterations and myocardial fibrosis. This stratification could potentially offer a deeper understanding and warrants exploration in future studies. We acknowledge the lack of coronary artery calcium scoring as a limitation. Although coronary artery calcium strongly correlates with atherosclerosis, our protocol primarily aimed at assessing myocardial fibrosis in relation to aortic stenosis and general cardiovascular risk factors [61].

The main strength of our study is the integration of routine CCT data with a rigorously validated ECV protocol, producing results that are directly transferable to everyday pre-TAVI workflows without additional scan time or radiation exposure. Future work should prospectively track ECV changes after valve replacement and expand enrollment across multiple centers to examine gender- and ethnicity-specific patterns and to clarify whether blood pressure and smoking cessation translate into measurable regression of diffuse fibrosis. Furthermore, future studies should assess myocardial ECV changes across different stages of cardiovascular–kidney–metabolic syndrome to better understand their combined impact on myocardial structure and function. Incorporating coronary artery calcium measurements will also be important to clarify the independent contributions of coronary atherosclerosis to myocardial extracellular volume, especially in smokers.

## 5. Conclusions

In patients scheduled for TAVI, AH, smoking, and smaller AVA are independently associated with increased left ventricular ECV as assessed by CCT. These observations require confirmation in prospective studies.

## Figures and Tables

**Table 1:** Baseline characteristics of the study population (*N* = 61).

**Quantitative Variables**					
**Variable**	**Mean**	**SD**	**Min**	**Max**	**Median**
Age [years]	78.82	9.15	44.00	91.00	82.00
Body mass index [kg/m^2^]	26.24	3.10	20.18	35.32	24.83
Systolic BP [mmHg]	135.49	31.59	90.00	230.00	140.00
Diastolic BP [mmHg]	78.28	13.99	60.00	100.00	90.00
Total cholesterol [mg/dL]	168.69	38.18	100.20	222.70	192.60
Triglycerides [mg/dL]	121.11	65.33	32.20	374.70	104.40
Glucose [mg/dL]	105.11	17.61	86.00	170.00	99.00
CVRF	3.61	1.38	1.00	7.00	3.00
**Qualitative Variables**					
**Variable**	**Absolute Value**		**Percentage**		
Gender					
Male	38		62.3		
Female	23		37.7		
Body mass index					
Normal body mass	33		54.1		
Overweight	22		36.1		
Obesity	6		9.8		
Arterial hypertension	33		54.1		
Hypercholesterolemia	35		57.4		
Hypertriglyceridemia	20		32.8		
Diabetes mellitus	6		9.8		
Smoking	28		45.9		

Abbreviations: BP, blood pressure; CVRF, cardiovascular risk factors; Max, maximum value; Min, minimum value; SD, standard deviation.

**Table 2:** Aortic valve assessment parameters and left ventricular myocardial extracellular volume were evaluated by multidetector cardiac computed tomography prior to transcatheter aortic valve implantation in the study population (*N* = 61).

**Variable**	**Absolute Value**		**Percentage**		
Number of aortic valve cusps					
Bicuspid valve (2 cusps)	2		3.3		
Tricuspid valve (3 cusps)	59		96.7		
**Variable**	**Mean**	**SD**	**Min**	**Max**	**Median**
AVCS	3321.91	1512.07	1095.70	7486.00	2975.00
Aortic annulus					
Max diameter [mm]	27.07	3.29	22.00	37.00	27.00
Min diameter [mm]	21.66	2.87	17.00	30.00	21.00
Mean diameter [mm]	24.36	2.76	20.50	32.00	24.00
Sinus of Valsalva					
Max diameter [mm]	33.84	4.07	27.00	46.00	33.00
Min diameter [mm]	30.72	4.27	19.00	45.00	30.00
Mean diameter [mm]	32.28	3.89	25.50	45.50	32.00
Height [mm]	20.54	3.01	14.00	30.00	20.00
Coronary ostial height from the annulus					
Left coronary artery [mm]	13.18	2.47	8.50	20.00	14.00
Right coronary artery [mm]	14.20	2.42	11.00	20.00	14.00
AVA [cm^2^]	0.99	0.17	0.74	1.48	0.95
ECV [%]	27.53	1.90	23.60	31.50	27.60

Abbreviations: AVA, aortic valve area; AVCS, aortic valve calcium score; ECV, extracellular volume; Max, maximum value; Min, minimum value; SD, standard deviation.

**Table 3:** Left ventricular myocardial extracellular volume assessed by multidetector cardiac computed tomography prior to transcatheter aortic valve implantation in the analyzed patient subgroups.

Variable		ECV [%] ^b^	*p*
Age ^a^	≥Me (≥82 years)	27.58 ± 1.89	ns.
	<Me (<82 years)	27.46 ± 1.94	
Gender	male	27.80 ± 1.92	ns.
	female	27.08 ± 1.82	
Obesity	yes	28.82 ± 0.88	ns.
	no	27.39 ± 1.94	
Arterial hypertension	yes	28.01 ± 1.83	0.03
	no	26.93 ± 1.85	
Hypercholesterolemia	yes	27.62 ± 1.80	ns.
	no	27.40 ± 2.06	
Hypertriglyceridemia	yes	27.63 ± 1.99	ns.
	no	27.48 ± 1.88	
Diabetes mellitus	yes	27.92 ± 2.12	ns.
	no	27.49 ± 1.89	
Smoking	yes	28.71 ± 1.41	0.01
	no	26.52 ± 1.68	
CVRF ^a^	≥Me (≥3)	27.96 ± 1.76	0.01
	<Me (<3)	25.95 ± 1.57	
AVCS ^a^	≥Me (≥2975.00)	28.08 ± 1.84	0.02
	<Me (<2975.00)	26.95 ± 1.82	
AVA ^a^	≥Me (≥0.95 cm^2^)	26.53 ± 1.65	0.01
	<Me (<0.95 cm^2^)	28.63 ± 1.53	

^a^ division into two subgroups based on the median of the given parameter; ^b^ mean ± standard deviation. Abbreviations: AVA, aortic valve area; AVCS, aortic valve calcium score; CVRF, cardiovascular risk factors; ECV, extracellular volume; Me, median; ns., not statistically significant.

**Table 4:** Correlations between left ventricular myocardial extracellular volume assessed by pre-procedural multidetector cardiac computed tomography and other quantitative variables in the study population (*N* = 61). Pearson correlation analysis.

Variable	ECV [%]	
	r	*p*
Age [years]	0.16	ns.
BMI [kg/m^2^]	0.30	0.01
Systolic BP [mmHg]	0.31	0.02
Diastolic BP [mmHg]	0.22	ns.
Total cholesterol [mg/dL]	0.06	ns.
Triglycerides [mg/dL]	0.24	ns.
Glucose [mg/dL]	0.21	ns.
CVRF	0.49	0.01
AVCS	0.36	0.01
AVA [cm^2^]	−0.59	0.01

Abbreviations: AVA, aortic valve area; AVCS, aortic valve calcium score; BMI, body mass index; BP, blood pressure; CVRF, cardiovascular risk factors; ECV, extracellular volume; ns., not statistically significant.

**Table 5:** Results of backward stepwise multiple linear regression using the ordinary least squares method: independent predictors of increased extracellular volume in the study population (*N* = 61).

Variable	Intercept	Arterial Hypertension ^1^	Smoking ^1^	AVA [cm^2^]
Regression coefficient β	30.114	1.218	1.575	−3.986
Standard error of the coefficient	1.374	0.344	0.412	1.213
*p*	0.001	0.003	0.004	0.002
*p* for the overall model	<0.001			
Adjusted R^2^ [%]	51.907			
Standard error of estimation	1.319			

^1^ Dichotomous variables coded as 0 = no, 1 = yes. Abbreviations: AVA, aortic valve area.

## Data Availability

The data presented in this study are available on request from the corresponding author (the data are not publicly available due to privacy or ethical restrictions).

## Abbreviations

The following abbreviations are used in this manuscript:

99mTc	Technetium-99m-labelled
AH	Arterial hypertension
AS	Aortic stenosis
ATTR-CA	Transthyretin cardiac amyloidosis
AVA	Aortic valve area
AVCS	Aortic valve calcium score
BMI	Body mass index
BP	Blood pressure
CAD	Coronary artery disease
CCT	Cardiac computed tomography
CMR	Cardiac magnetic resonance
CVRF	Cardiovascular risk factors
DM	Diabetes mellitus
DPD	99mTc-3,3-diphosphono-1,2-propanodicarboxylic acid
ECV	Extracellular volume
HF	Heart failure
HMDP	99mTc-hydroxymethylene diphosphonate
HU	Hounsfield units
PYP	99mTc-pyrophosphate
ROI	Regions of interest
SAVR	Surgical aortic valve replacement
TAVI	Transcatheter aortic valve implantation
